# Effect of H2S on endothelial barrier function impairment in anorectal vascular plexus caused by deoxycholic acid

**DOI:** 10.3389/fmed.2025.1537723

**Published:** 2025-09-04

**Authors:** Han Yan, Ya-Lun Li, Shao-Rong Pan, Yuan-Yuan Ma, Jing Zhu, Peng-Yuan Wang, Ze-Yang Chen

**Affiliations:** ^1^Endoscopy Center, Peking University First Hospital, Beijing, China; ^2^Department of Gastrointestinal Surgery, Peking University First Hospital, Beijing, China

**Keywords:** GYY4137, deoxycholic acid, hemorrhoids, anorectal vascular plexus, vascular endothelial barrier function

## Abstract

**Introduction:**

As a vascular-related disease, hemorrhoids cause pathological changes, such as abnormal dilation in the anorectal vascular plexus (AVP), which may be closely related to injury to vascular endothelial barrier function (VEBF). Elevated deoxycholic acid caused by a high-fat diet can impair intestinal barrier function. However, the effect of VEBF impairment in AVP caused by DCA is unclear. The aim of our study was to investigate the effects of DCA and GYY4137 on the VEBF in AVP and to explore the pathogenesis of hemorrhoids and new treatment ideas.

**Methods:**

A HUVECs monolayer model and mouse model were generated with a high DCA concentration and used to investigate the effect of GYY4137 on SDC-induced VEBF disruption in AVP and the underlying mechanism.

**Results:**

In the HUVECs monolayer model, DCA significantly increased the permeability of the monolayer and altered the distribution of tight junction proteins (TJPs) by increasing the levels of myosin light chain kinase and myosin light chain phosphorylation. GYY4137 pretreatment significantly improved DCA-induced VEBF dysfunction. GYY4137 can also increase resistance to SDC-induced VEBF injury and improve the distribution of TJPs in the AVP in mouse model.

**Conclusion:**

GYY4137 can improve the distribution of TJPs by inhibiting the activation of the MLCK-P-MLC2 signaling pathway induced by DCA, thereby protecting the VEBF in AVP, which may be applied to hemorrhoids therapy in the future.

## Introduction

Hemorrhoids is one of the common diseases of the anorectal system (1), and its prevalence varies in different regions of the world, ranging from 14 to 39% (2–4). Hemorrhoids are commonly associated with bleeding, swelling, mild discomfort, and anal itching (5). The anorectal vascular plexus (AVP) is the submucosal vascular plexus in the anorectal region. In patients with hemorrhoids, the AVP is usually located in the submucosa of the lesion, also known as the “rectal cavernous body” or “hemorrhoidal vascular plexus” (6). The endoscopic appearance of AVP often manifests as dilated and tortuous blood vessel clusters (7), and recent studies have shown that AVP in hemorrhoidal patients can cause pathological changes, such as increased blood flow, abnormal dilatation of the blood vessel wall, obvious angiogenesis and edema (8), which may be closely related to vascular endothelial barrier function (VEBF) injury, representing a vascular-related disease.

Studies have shown that a high body mass index is a common risk factor for hemorrhoids (9), especially when individuals are consumed a high-fat diet, which plays an important role in the development of hemorrhoids (10). Hyperlipidemia can increase the concentration of deoxycholic acid (DCA) in the intestine and blood circulation (11). DCA is a major component of hydrophobic secondary bile acids, and its cytotoxicity and intestinal barrier destruction have been well reported (12). It has been shown that DCA or sodium deoxycholate (SDC) induces changes in the expression and localization of tight junction proteins (TJPs) in the intestinal epithelium, leading to impairment of intestinal epithelial barrier function (13). TJPs are also the main connecting components among vascular endothelial cells and play an important role in maintaining the permeability and integrity of vessel walls (14). However, there are no relevant thorough studies on the effect of elevated DCA induced by hyperlipidemia on VEBF in hemorrhoidal AVP.

Endogenous H2S (hydrogen sulfide), a gas signaling molecule, is considered to be the third most important gas signaling molecule after nitric oxide and carbon monoxide (15) and is involved in a variety of physiopathological processes, such as anti-inflammation (15) and vasodilation (16). Recent studies have shown that GYY4137, an H2S donor, can antagonize the reduced expression of TJPs in the intestinal epithelium induced by endotoxin (17) and can alleviate the disorder of TJPs arrangement caused by TNF/IFN by inhibiting the activation of the MLCK-P-MLC2 pathway, thus protecting the function of the intestinal mucosa (18). However, whether H2S can protect TJPs in the hemorrhoidal vascular endothelium is unclear.

In this study, we investigated the disruptive and protective effects of SDC and GYY4137 on anorectal VEBF from cellular, animal, and human perspectives. Our study may provide new insights for exploring the pathogenesis of hemorrhoids and provide new ideas for subsequent treatment of hemorrhoids triggered by SDC-related etiologies.

## Materials and methods

### Primary reagents

SDC, GYY4137, and FD-40 were purchased from Sigma–Aldrich Corporation, USA.

The primary antibodies used for Western blotting and immunofluorescence were as follows: ZO-1 (Invitrogen, Thermo Fisher Scientific Inc., USA), Occludin (Invitrogen, Thermo Fisher Scientific Inc., USA), MLC2 (ABclonal Biotechnology Co., Ltd. China), P-MLC2 (Cell Signaling Technology, Inc. China), and MLCK (Abcam, China).

The following secondary immunofluorescence antibodies were used: Alexa Fluor 488-conjugated goat anti-rabbit IgG (H + L) (Thermo Fisher Scientific Inc., USA) and Alexa Fluor 647-conjugated goat anti-mouse IgG (H + L) (Thermo Fisher Scientific Inc., USA).

The primary antibodies used for immunohistochemistry were as follows: biotin-conjugated mouse IgG polyclonal antibody (Proteintech Group, Inc. China); Polyclonal antibody for MPO (Proteintech Group, Inc. China).

### Cell experiment

#### Cell culture

Human umbilical vein endothelial cells (HUVECs) were used as experimental cells. The cells were cultured in DMEM (Dulbecco’s modified Eagle’s medium) supplemented with 10% fetal bovine serum, penicillin–streptomycin mixture (x100) and 2 mM L-glutamine. HUVECs were cultured at a density of 10^5^–10^6^ cells/well in transwell plates (12-well, 12 mm, 0.4 μm), 6-well plates, and 24-well plates with cell crawlers. After the cells were completely confluent, the Transwell plates were administered GYY4137 in the lower chamber and SDC in the upper chamber. GYY4137 was added to 6-well and 24-well plates first, followed by SDC. SDC was used to treat cells for 2 h at a concentration of 0.4 mmol/L (see below in the RESULTS section); GYY4137 was used to treat cells for 48 h at a concentration of 200 μmol/L (this concentration and duration were based on a previous study by our group (19)).

### Measurement of the transepithelial electrical resistance (TEER) of vascular endothelial cells

The TEER of each well was measured before and after reagent administration using a Millocell ERS-2 resistance meter (World Precision Instrument, Inc., USA), and each well was measured 3 times at different positions to obtain the average value. Three replicate wells were set up for each group.

### Permeability of FD-40 in a monolayer cell model

Before and after reagent administration, the monolayer was rinsed with phosphate buffered saline (PBS) solution on a Transwell plate, and 1 mg/mL FD-40 solution diluted with Hank’s equilibrium salt solution was added to the upper chamber for 1 h. 100 μL of the solution was taken from the lower chamber, and the absorbance of FD-40 was measured at an excitation wavelength of 492 nm and an emission wavelength of 520 nm using a Synergy H1 Hybrid Reader. At the same time, FD-40 was diluted at a continuous ratio, and an absorbance-concentration standard curve was drawn to determine the concentration of FD-40.

#### Western blot

Total protein was extracted from HUVECs seeded in 6-well plates, and the protein concentration was determined via the BCA method. Protein samples were added to each well of the precast gel for electrophoresis, and the separated proteins were transferred to a PVDF membrane. The membrane was blocked with 5% BSA for 1.5 h at room temperature, after which the primary antibody was added, and the mixture was incubated at 4 °C overnight. Subsequently, the membrane was incubated with the secondary antibody (1:5000 dilution) at room temperature for 1 h. Finally, the membrane was imaged with an enhanced chemiluminescence (ECL) reagent on a GeneGnome imaging system.

#### Immunofluorescence of ZO-1 and Occludin in HUVECs monolayer cell models

6-well plates were discarded from the medium, rinsed with PBS, added with methanol at −20 °C overnight, fixed with acetone at −20 °C for 1 min, then blocked with 1% BSA for 1 h at room temperature, incubated with mouse anti-human Occludin (1:150) and rabbit anti-human ZO-1 (1:150) primary antibody working solution overnight at 4 °C, and then incubated with Goat Anti Mouse IgG (H&L)-Alexa Flour 488 and Goat Anti Rabbit IgG (H&L)-Alexa Flour 647 secondary antibody working solution at room temperature, protected from light for 1 h. Finally, the slices were blocked with DAPI and stored in the dark. Fluorescence was observed under a confocal microscope.

### RT–PCR

RNA was extracted with chloroform as an alternative, isopropyl alcohol, and an RNA solubilizer. A reverse transcription reaction system was prepared, which were gently mixed and centrifuged. Reverse transcription was completed on the PCR instrument. The products were completely amplified on a fluorescent quantitative PCR instrument. (detailed procedure in Table 1).

**Table 1:** Detailed procedure of RT-PCR test conditions.

	Component	Volume
Reverse transcription reaction system	5 × SweScript All-in-One SuperMix for qPCR	4 μL
	gDNA Remover	1 μL
	Total RNA ^*^	10 μL
	RNase free water	Add to 20 μL

	Temperature	Time
Reverse transcription	25 °C	5 min
	42 °C	30 min
	85 °C	5 s

PCR amplification		
Stage 1	Stage 2 (40 cycles)	Stage 3 (Melting curve)
95 °C, 30 s pre-denaturation	95 °C, 15 s denaturation	65 °C → 95 °C
	60 °C, 30s annealing / extension	Acquire fluorescence signal once for every 0.5 °C of temperature increase.

### Animal experiment

#### Animals

Male C57BL/6 mice (4 weeks old) were purchased from SiPeiFu and housed in the isolation unit of the Laboratory Animal Center of Peking University First Hospital. For the SDC treatment, an aqueous solution of 0.2% SDC was used to feed the mice; for the GYY4137 treatment, the mice were injected intraperitoneally with 50 mg/(kg/d) GYY4137 twice a week for 3 weeks before sacrifice. The subgroups were as follows: control group (no treatment with SDC or GYY4137), SDC group (treatment with SDC only), GYY4137 group (treatment with GYY14137 only), and SDC + GYY4137 group (treatment with both SDC and GYY4137). The mice were sacrificed after a total of 12 weeks. After sacrifice, the anorectal tissues were removed from the mice, frozen and fixed for subsequent experiments. All procedures were approved by the Ethics Committee of Peking University First Hospital (approval No. J2022096).

### IgG immunohistochemistry of mouse anorectal tissue

Mouse tissues were routinely sliced and then antigenically retrieved with sodium citrate antigen repair solution (pH 6.0) in a microwave oven. The slices were blocked with an endogenous myeloperoxidase blocker for 10 min and then sealed with 1% BSA at 36.8 °C for 1 h. Afterward, the sections were incubated with a Biotin-conjugated Mouse-IgG Polyclonal antibody (1:200) overnight at 4 °C. Then, the sections were incubated with enzyme-labeled goat anti-mouse/rabbit IgG polymer for 30 min at room temperature. Then, the sections were stained with DAB reagent for 45 s and hematoxylin for 3 min. The slices were sequentially placed in 70, 85, 95, and 100% alcohol and xylene for transparency. The slices were sealed with neutral resin. Three perivascular fields of the same size were selected from each group, and the number of positive IgG signals was counted.

#### Immunofluorescence of ZO-1 and Occludin in mice anorectal tissues

Mouse tissues were routinely sliced and then antigenically repaired with EDTA antigen repair solution (pH 8.0) in a microwave oven. The slices were incubated with rabbit anti-human ZO-1 (5 μg/mL) and mouse anti-human Occludin (3 μg/mL) primary antibody working solution overnight at 4 °C and then incubated with Alexa Fluor 488-conjugated goat anti-rabbit IgG (H + L) (4 μg/mL) and Alexa Fluor 647-conjugated goat anti-mouse IgG (H + L) (4 μg/mL) at 36.8 °C in the dark for 1 h. DAPI was added, and the samples were observed under a confocal microscope.

#### MPO immunohistochemistry of mouse anorectal tissue

The primary antibody was incubated with MPO (1:100). The rest of the procedure was the same as that for IgG immunohistochemistry. Assessment method: Three visual fields were selected according to the degree of staining from light to deep and were assigned scores of 1, 2, and 3. Three visual fields were selected from each group, and each field was scored by three medical workers with a pathological background. The average score was taken as the staining score of the field.

#### Human tissue experiment

##### Immunofluorescence of ZO-1 and Occludin in human anorectal tissues

Surgical excision specimens from 3 patients with mixed hemorrhoids of clinical grade III-IV who underwent “external peeling and internal ligation” and 3 normal anorectal tissue specimens from 3 patients without hemorrhoidal disease who underwent “anorectal resection” for other diseases were subjected to TJP immunofluorescence. The method used for mouse tissue immunofluorescence was the same as that described above. The patients have signed the informed consent, and the experiment has been approved by the Ethics Committee of Peking University First Hospital.

### Statistical analysis

The measurement results are expressed as the mean ± standard error of the mean (SEM) and were analyzed for differences using t tests and one-way analysis of variance (ANOVA). *p* < 0.05 was considered to indicate statistical significance. Statistical analysis was performed using GraphPad Prism 8.0 software.

## Results

### Effects of GYY4137 and SDC on monolayer cell models of HUVECs

#### Effect of SDC on TEER values

**Figure 1:**
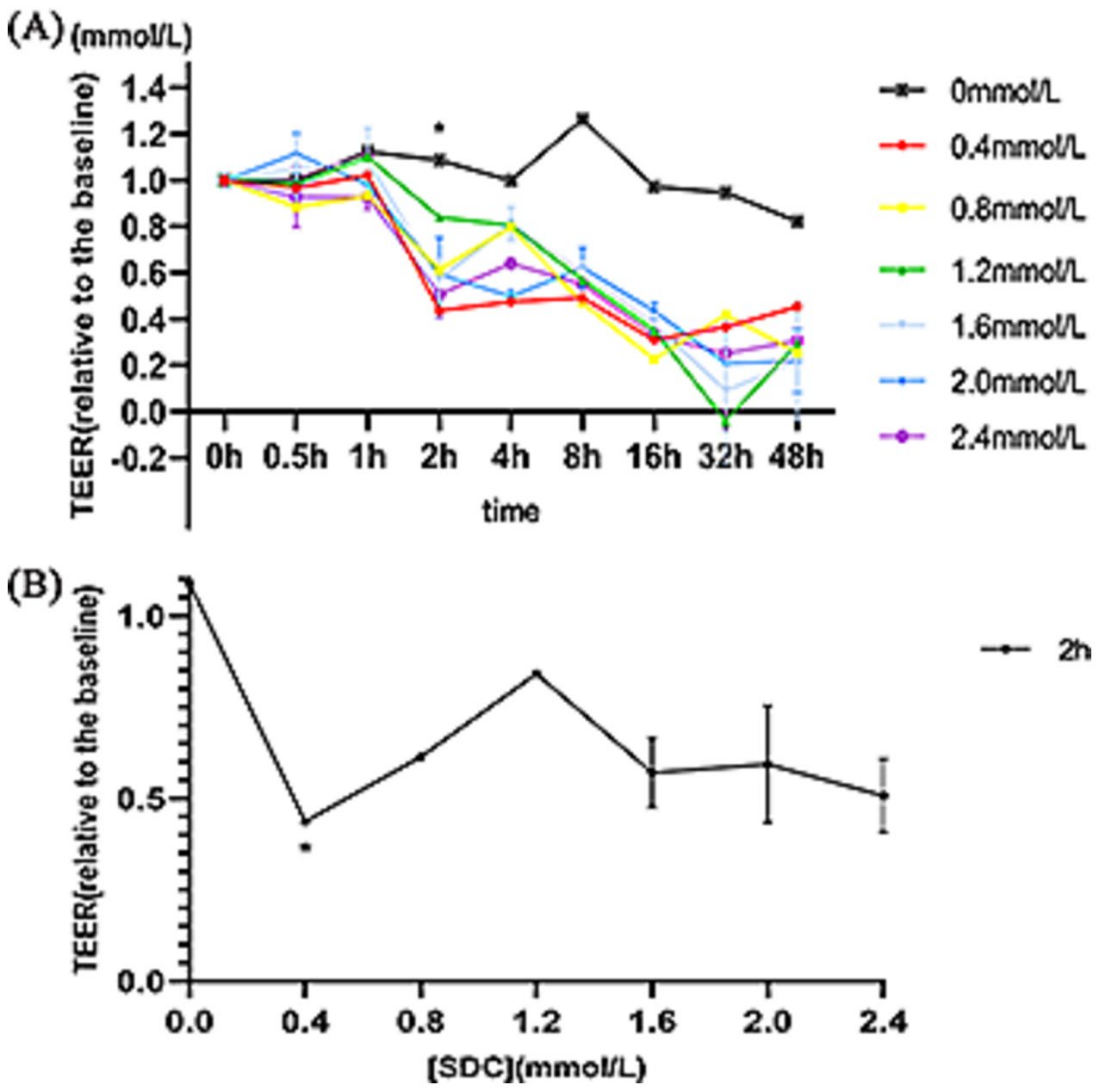
The TEER of different concentrations of SDC at different times. **(A)** Different concentrations of SDC (0.4–2.4 mmol/L) were used to treat HUVEC monolayers at different times (0–48 h). TEER decreased significantly when the processing time reached 2 h. **(B)** The TEER of cells treated with different concentrations of SDC for 2 h. At 2 h, the TEER decreased significantly in all the groups, but there was no significant difference among the different concentration groups. * Compared with that in the control group (SDC concentration was 0), the TEER in the treatment group was significantly lower (*p* < 0.05).

DMEM was used to dilute SDC at different concentrations (0 mmol/L, 0.4 mmol/L, 0.8 mmol/L, 1.2 mmol/L, 1.6 mmol/L, 2.0 mmol/L, and 2.4 mmol/L). 0.5 mL DCA was applied on HUVECs monolayer cells in the upper chamber of transwell at different times (0 h, 0.5 h, 1 h, 2 h, 4 h, 8 h, 16 h, 32 h, 48 h), and TEER values were measured at the corresponding time periods. As shown in Figure 1A, the TEER did not significantly change with respect to the concentration of SDC within 0–1 h. At 2 h, the TEER decreased significantly, but no significant decrease was observed for a specific concentration of DCA. As shown in Figure 1B, compared with that in the initial state, the TEER in each concentration group decreased significantly at 2 h, but the difference among the different concentration groups was not obvious. To ensure that the cells maintained good activity, we selected the lowest concentration and the shortest action time that caused the most significant decrease in the TEER to construct the vascular endothelial cell injury model; i.e., 0.4 mmol/L SDC acted on the monolayer of HUVECs for 2 h.

#### Protective effect of GYY4137 against SDC-induced barrier injury

**Figure 2:**
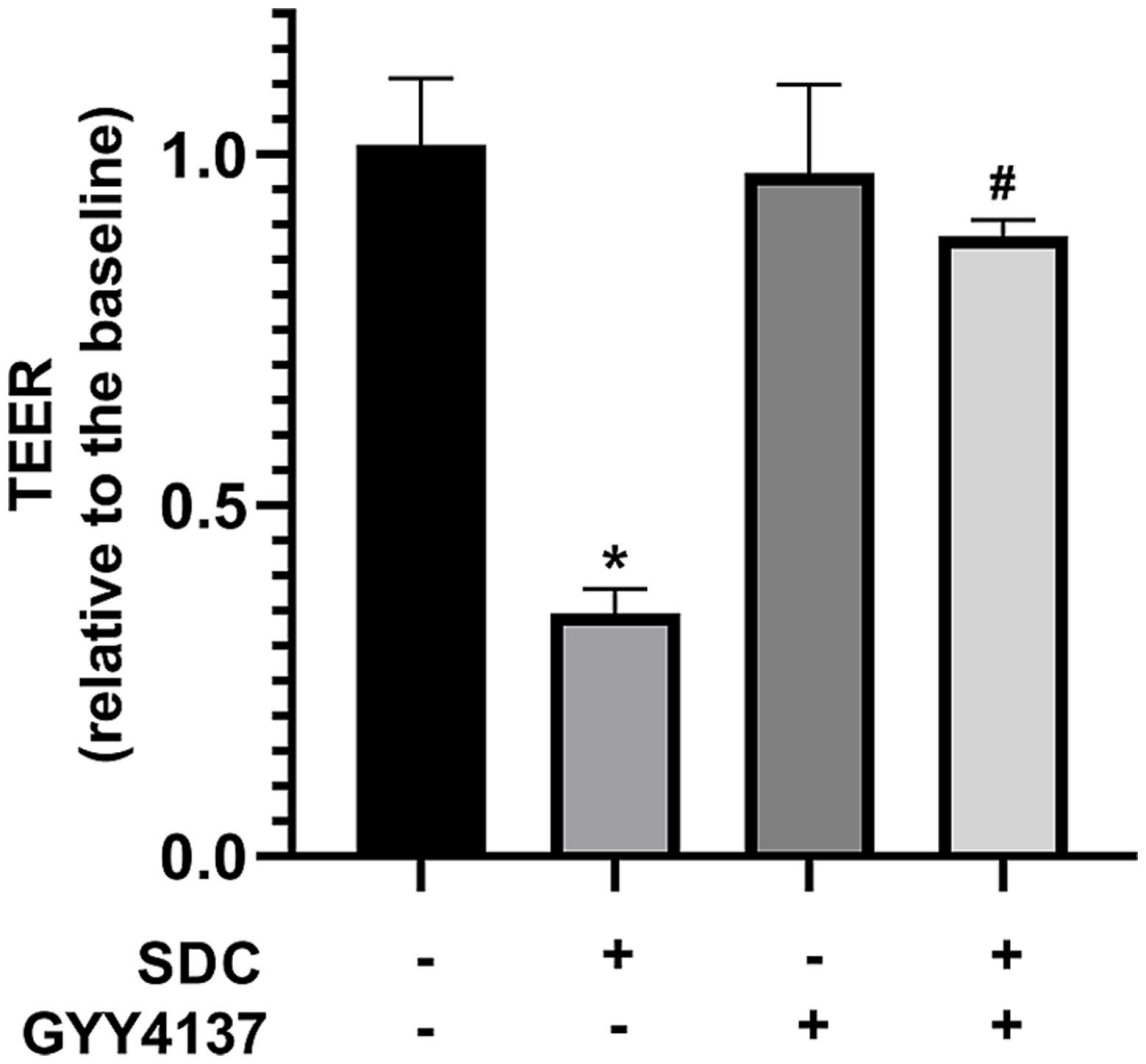
The TEER of different groups. Compared with that in the control group, the TEER in the SDC group was significantly lower. Compared with that in the SDC group, the TEER in the SDC + GYY4137 group was significantly greater. * vs. control group, *p* < 0.05; # vs. SDC group, *p* < 0.05.

The TEER of each group was measured, and the changes in TEER before and after administration were compared to determine the changes in VEBF in the endothelial monolayer cell model. As shown in Figure 2, the TEER of the SDC group decreased significantly, after GYY4137 was added, the TEER increased.

**Figure 3:**
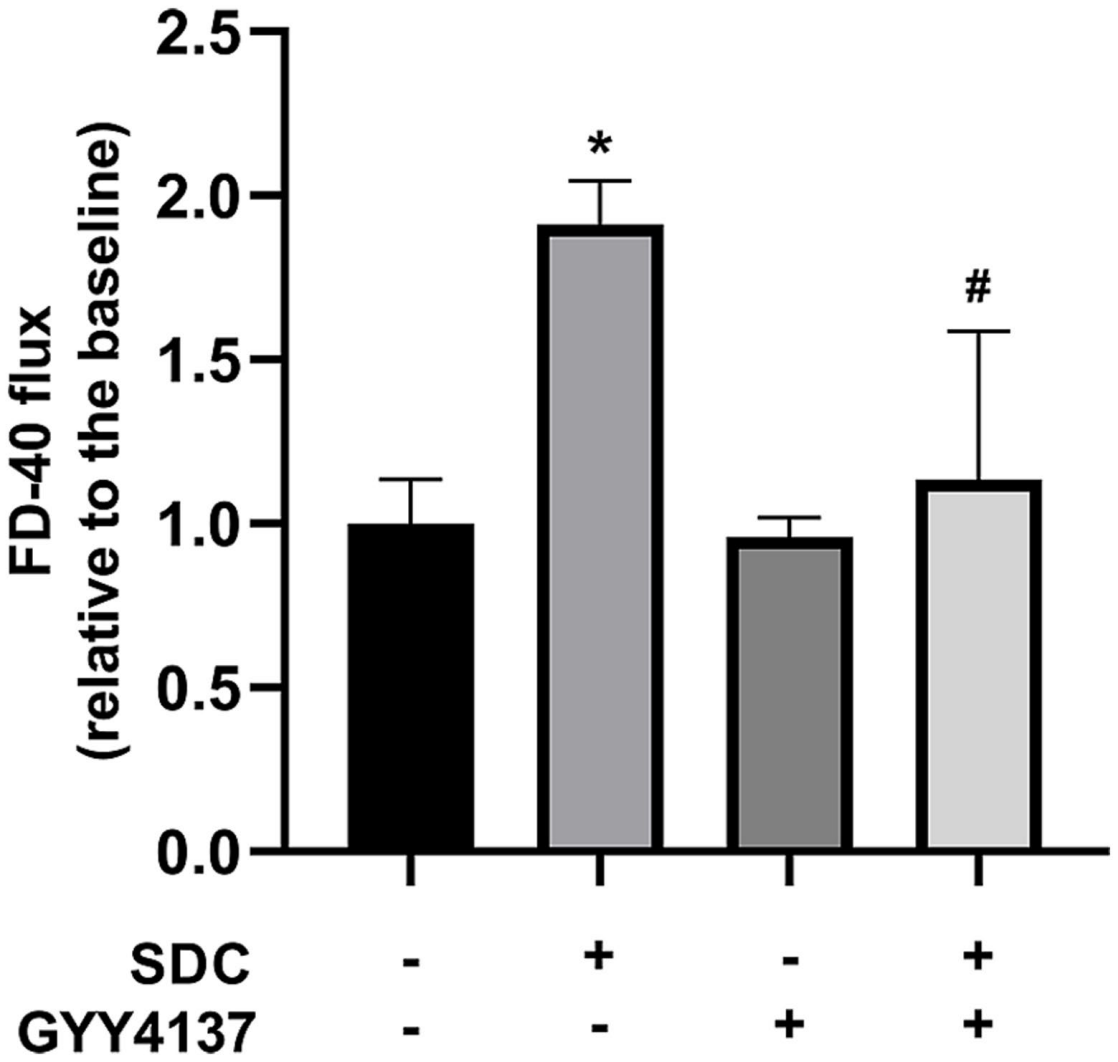
The FD-40 of different groups. Compared with that in the control group, FD-40 permeability was significantly greater in the SDC group. Compared with that in the SDC group, the FD-40 permeability in the SDC + GYY4137 group was significantly lower. * vs. control group, *p* < 0.05; # vs. SDC group, *p* < 0.05.

The concentration of FD-40 in the lower transwell chamber was measured before and after administration, and the change trend reflected the change in barrier function in the vascular endothelial monolayer model. As shown in Figure 3, compared with that in the control group, the FD-40 permeability in the SDC group was significantly greater, and the FD-40 permeability was significantly lower after GYY4137 was added.

#### Effects of GYY4137 and SDC on TJPs

**Figure 4:**
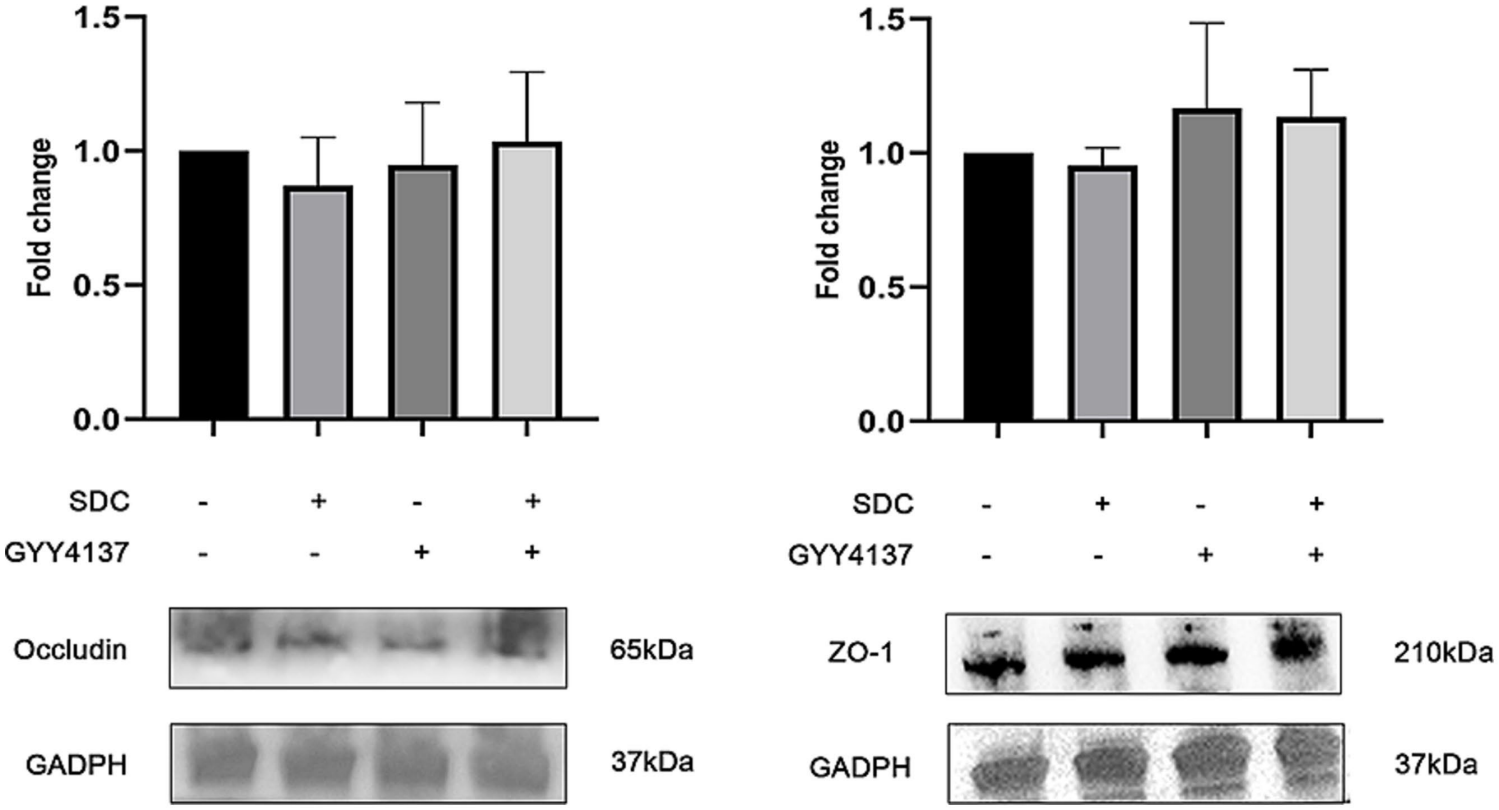
The expression of Occludin and ZO-1 in different groups. Compared with those in the control group, the expression of Occludin and ZO-1 in the SDC group was slightly lower. Compared with those in the SDC group, the expression of Occludin and ZO-1 in the SDC + GYY4137 group was slightly greater. However, the differences were not statistically significant.

Western blotting was performed on two TJPs, ZO-1 and Occludin, to assess their expression. As shown in Figure 4, compared with those in the control group, the expression of Occludin and ZO-1 in the SDC group was slightly lower; however, the expression of Occludin and ZO-1 in the GYY4137 + SDC group was slightly greater than that in the SDC group, but the differences were not statistically significant.

**Figure 5:**
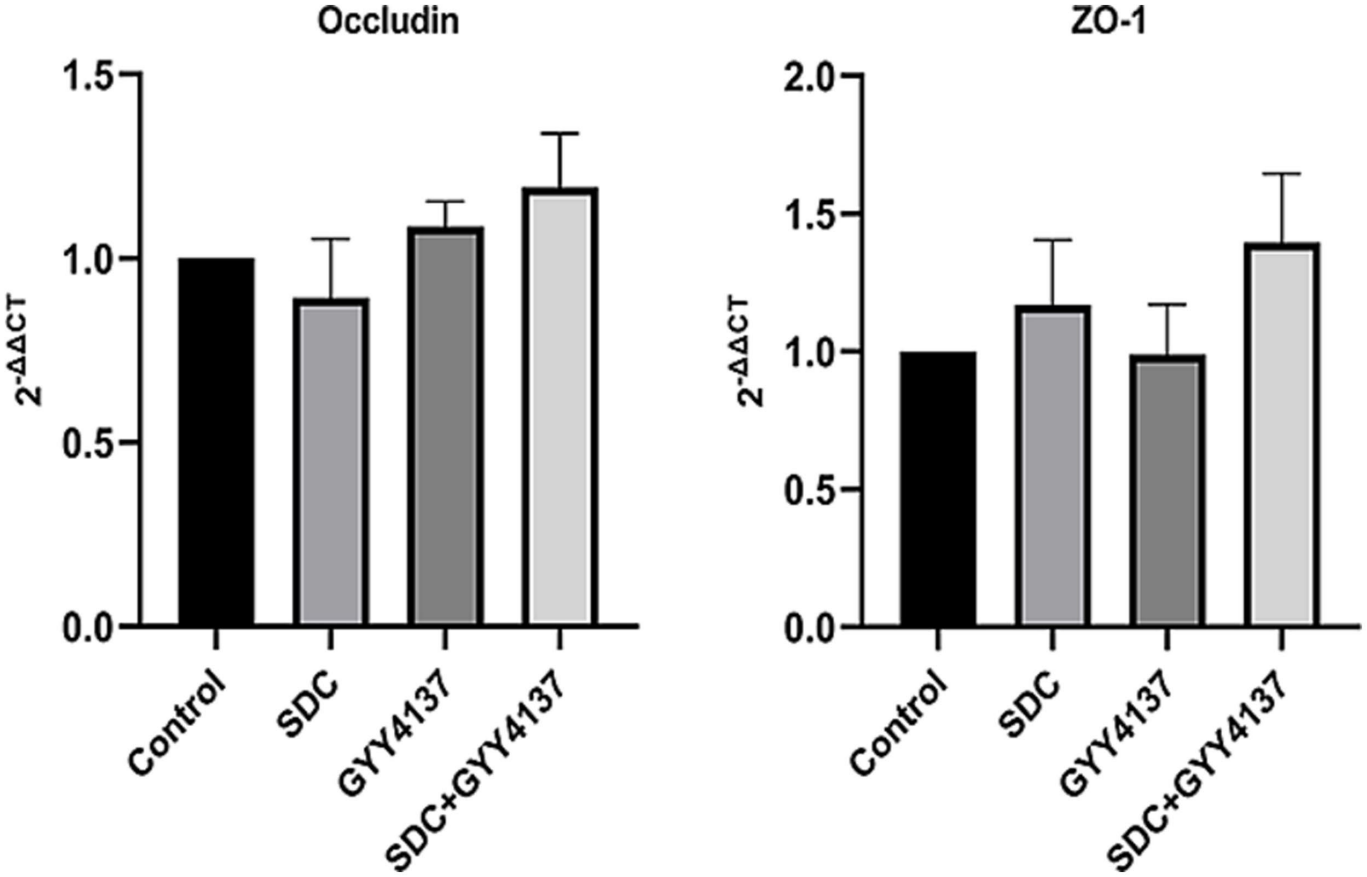
The RT-PCR of Occludin and ZO-1 in different groups. mRNA levels of Occludin and ZO-1 did not significantly differ among the groups.

RT–PCR was performed on Occludin and ZO-1, and the results are shown in Figure 5. The results indicated that there was no significant difference in Occludin or ZO-1 mRNA expression among the groups.

**Figure 6:**
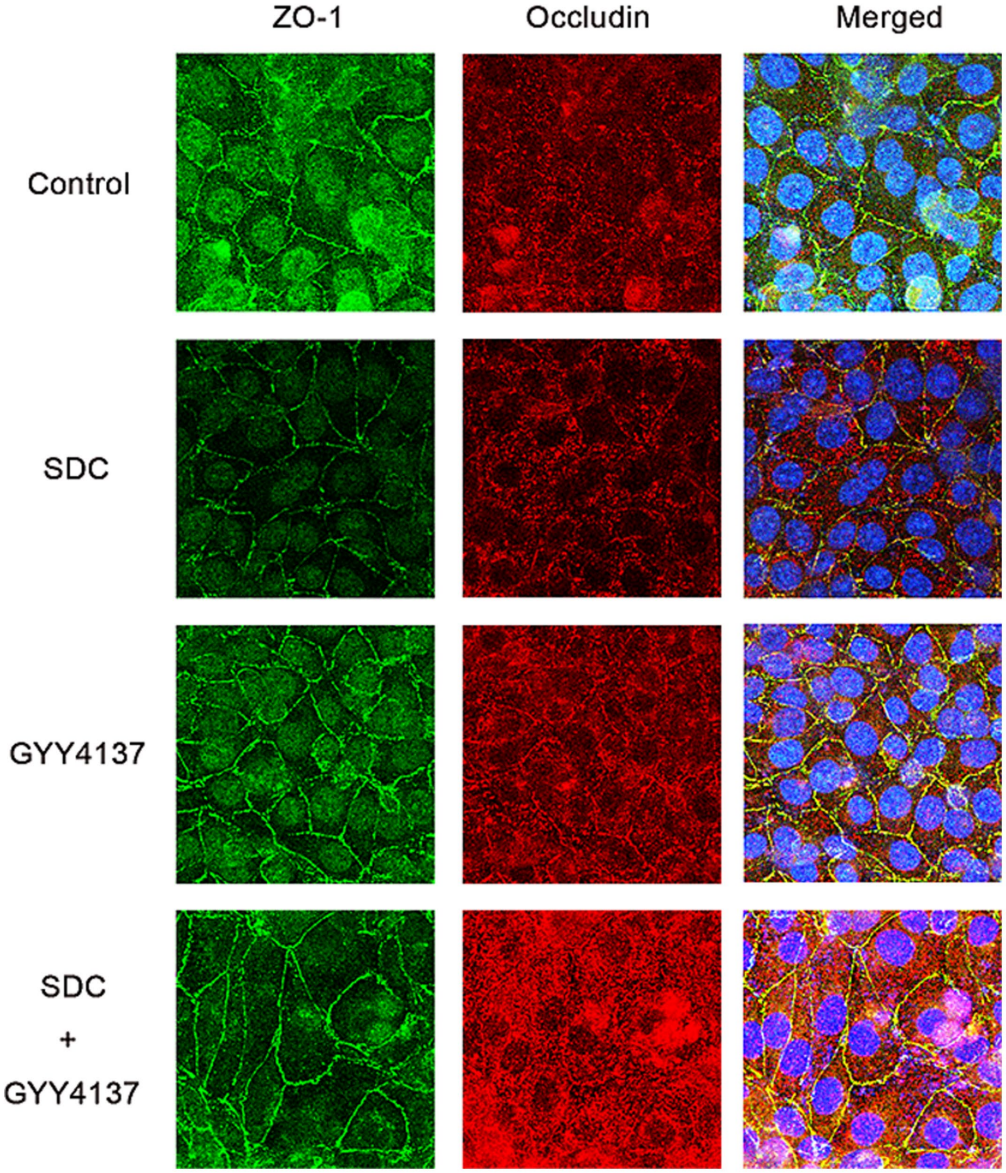
The fluorescence of Occludin and ZO-1 in different groups. Compared with those in the control group, the fluorescence continuity of Occludin and ZO-1 in the SDC group was decreased, and the fluorescence intensity was decreased. Compared with that in the SDC group, the fluorescence continuity in the SDC + GYY4137 group was improved, and the fluorescence intensity was restored.

The distribution of ZO-1 and Occludin was tested by immunofluorescence. As shown in Figure 6, compared with those in the control group, the fluorescence of ZO-1 and Occludin in the SDC group was wrinkled and discontinuous, and their brightness was reduced. After the addition of GYY4137, the fluorescence profile recovered, and the fluorescence intensity increased. These indicates that SDC can disorganize the distribution of TJPs, while GYY4137 can antagonize the damage caused by SDC.

#### Effects of GYY4137 and SDC on the activation of the MLCK-P-MLC2 signaling pathway

**Figure 7:**
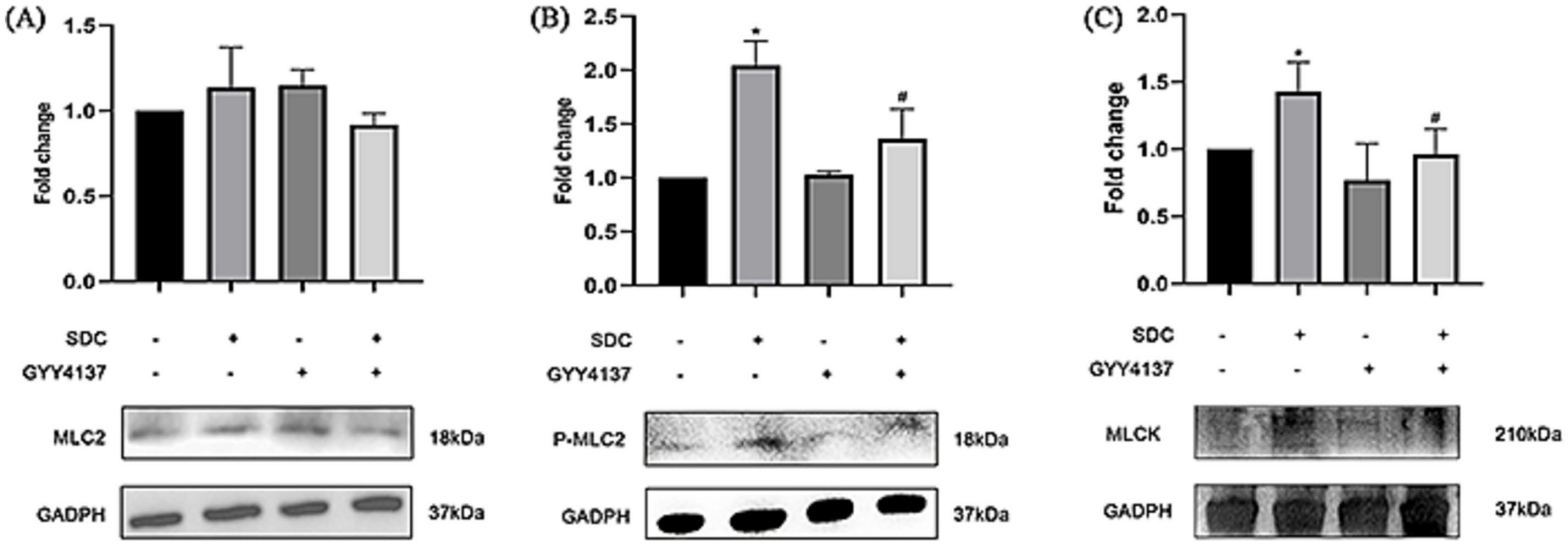
The expression of MLCK-P-MLC2 in different groups. **(A)** No significant difference in the expression level of MLC2 was found among the groups. **(B)** Compared with that in the control group, P-MLC2 expression was significantly greater in the SDC group. Compared with that in the SDC group, P-MLC2 expression was lower in the GYY4137 + SDC group. **(C)** The MLCK level in the SDC group was significantly greater than that in the control group. Compared with that in the SDC group, the MLCK expression in the GYY4137 + SDC group was decreased (* vs. the control group, *p* < 0.05; # vs. the SDC group, *p* < 0.05).

Western blotting was performed for MLC2, P-MLC2 and MLCK. As shown in Figure 7, there was no significant difference in the expression of MLC2 among the groups. However, P-MLC2 and MLCK were significantly increased in the SDC group, and P-MLC2 and MLCK were significantly decreased after GYY4137 was added.

### Effects of GYY4137 and SDC on the vascular endothelium of anorectal tissue in mice

#### Effects of GYY4137 and SDC on permeability

**Figure 8:**
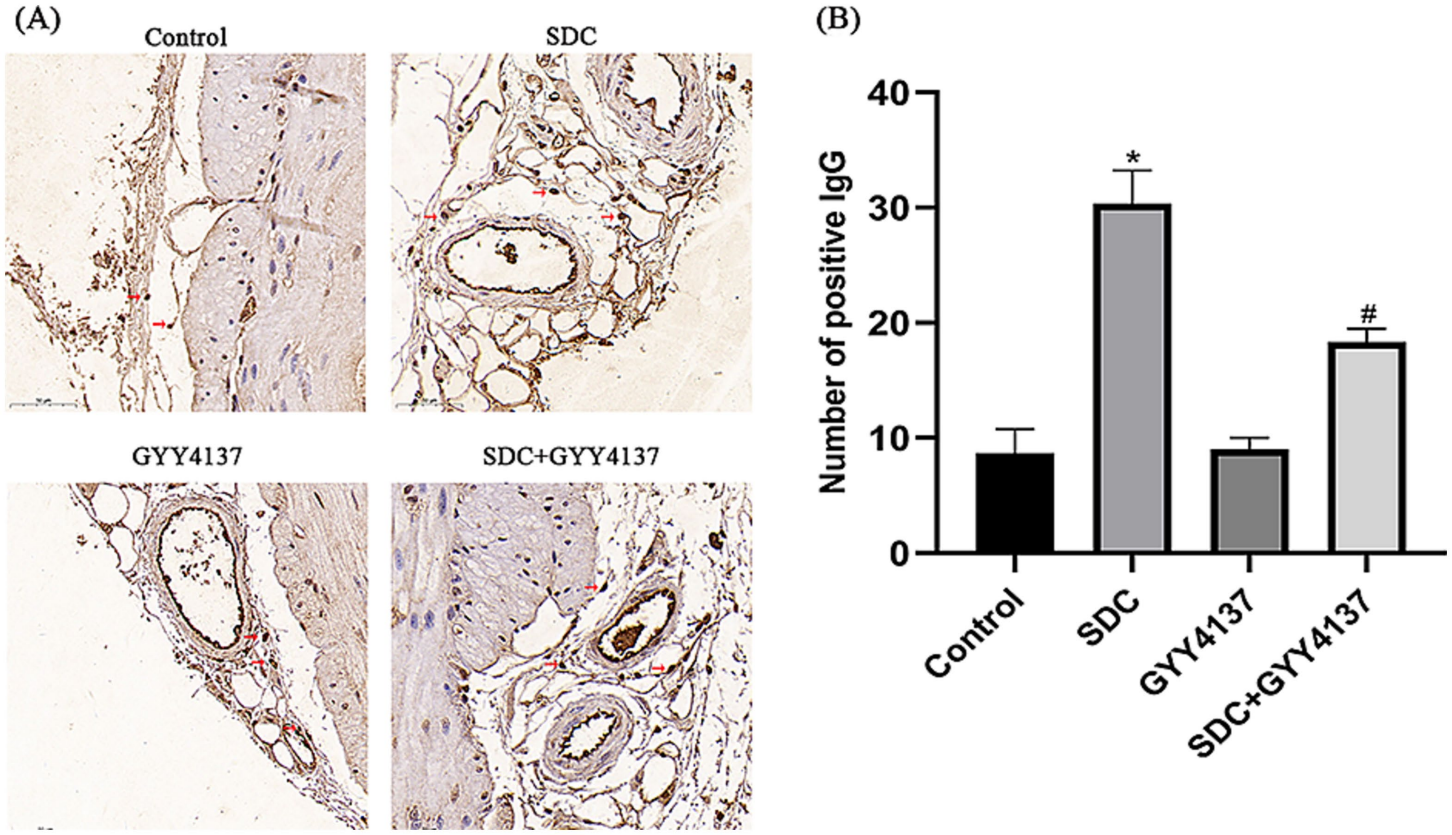
IgG immunohistochemical staining of perivascular anorectal tissue in mice. **(A)** Compared with that in the control group, the number of perivascular IgGs was significantly greater in the SDC group and significantly lower after GYY4137 was added. (The arrows indicate partial positive IgG signals) **(B)** * vs. the control group, *p* < 0.05; # vs. the SDC group, *p* < 0.05.

IgG immunohistochemistry can reflect the VEBF, and the more IgG exudates around blood vessels, the higher the vascular permeability. The results are shown in Figure 8. With the same visual field size, a small amount of IgG was observed around blood vessels in the control group and GYY4137 group. IgG staining was significantly increased in the SDC group. The perivascular IgG concentration was significantly lower in the SDC + GYY4137 group than in the SDC group. These findings indicated that the AVP VEBF phenotype of SDC-treated mice was significantly impaired, while GYY4137 significantly alleviated SDC-induced VEBF damage.

#### The impact of GYY4137 and SDC on TJs

**Figure 9:**
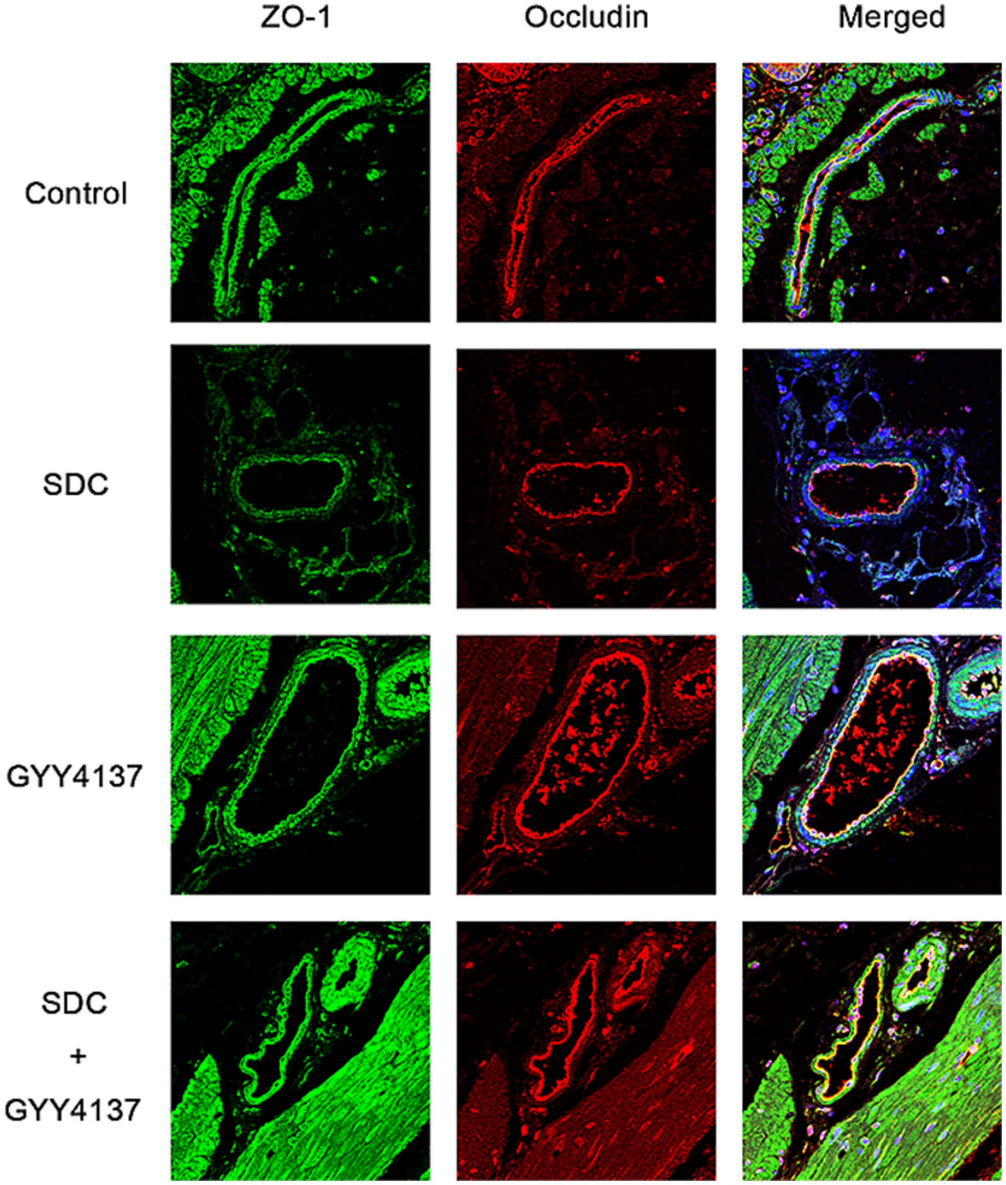
Immunofluorescence staining of ZO-1 and Occludin in mouse AVP tissue. Compared with those in the control group, the continuity of Occludin and ZO-1 was discontinuous, and the fluorescence intensity was weakened in the SDC group; compared with those in the SDC group, the continuity of Occludin and ZO-1 was greater, and the fluorescence intensity was restored in the SDC + GYY4137 group.

Immunofluorescence staining for Occludin and ZO-1 was performed, and the results are shown in Figure 9. Compared with those in the control group, the fluorescence of both Occludin and ZO-1 in the SDC group was wrinkled and discontinuous, and the fluorescence intensity was significantly weakened. However, compared with that in the SDC group, the fluorescence continuity in the SDC + GYY4137 group was significantly strengthened, and the fluorescence intensity was significantly restored. This suggests that SDC disrupts the distribution of TJPs, whereas GYY4137 antagonizes the SDC-induced distribution abnormalities.

#### Effects of GYY4137 and SDC on inflammation

**Figure 10:**
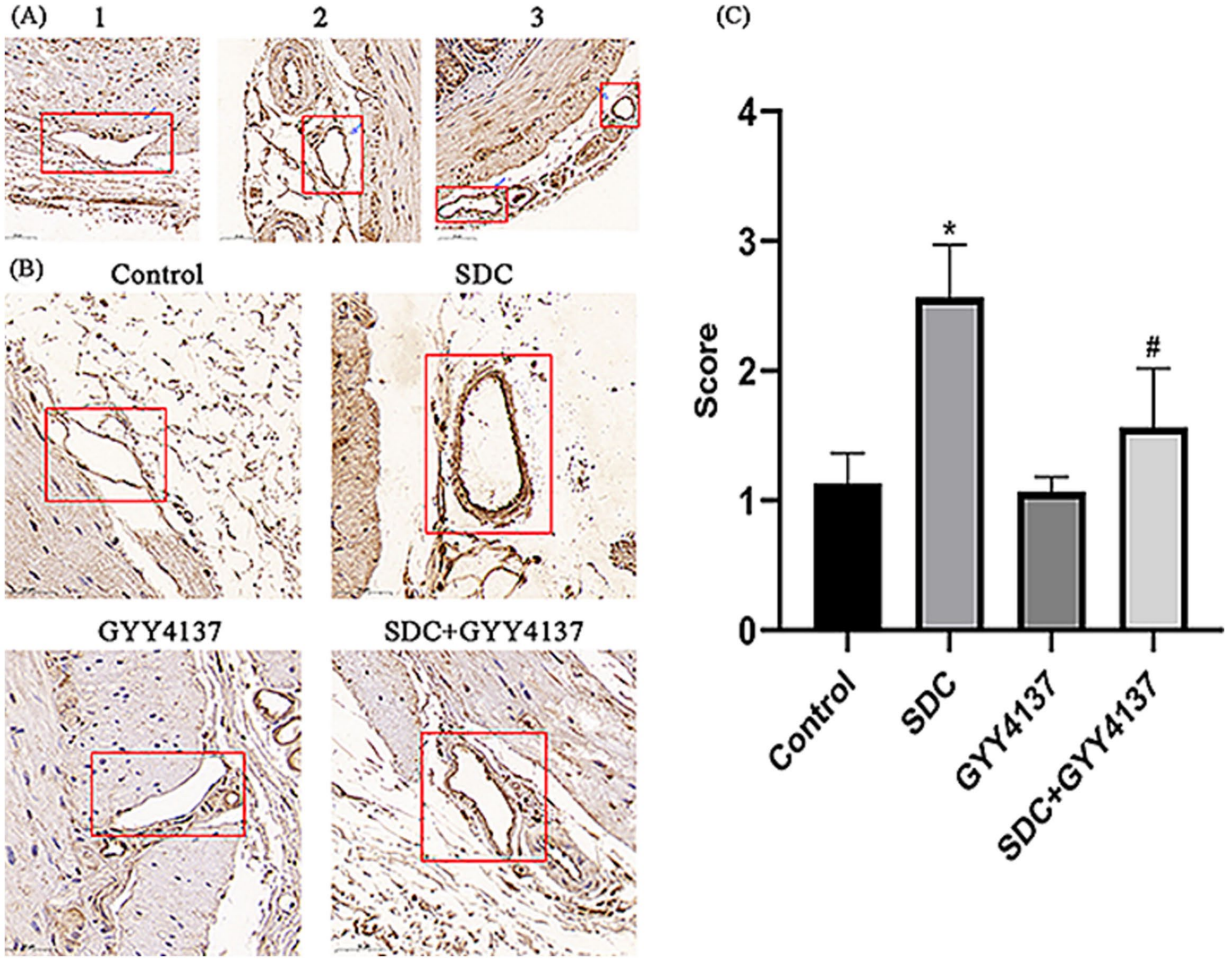
Immunohistochemical staining of MPO in the vascular wall of mouse AVP. **(A)** Different staining intensities were defined as 1, 2 and 3 points. **(B)** Compared with that in the control group, the blood vessel wall MPO in the SDC group was significantly deeper, and the MPO activity was significantly weakened after GYY4137 was added. **(C)** * vs. the control group, *p* < 0.05; # vs. the SDC group, *p* < 0.05.

MPO is an indicator of inflammation, which often accumulates in the walls of blood vessels. The results of MPO immunohistochemistry are shown in Figure 10. A small amount of MPO staining was observed in the vascular wall of the control group and the GYY4137 group, indicating that there was no obvious inflammation in the blood vessels; in the SDC group, there was a significant increase in MPO staining; and in the SDC + GYY4137 group, MPO staining was significantly weakened compared with that in the SDC group, indicating that inflammation in the blood vessels was alleviated.

### Effects of GYY4137 and SDC on AVP in patients with hemorrhoids and normal controls

#### Effects of GYY4137 and SDC on TJPs

**Figure 11:**
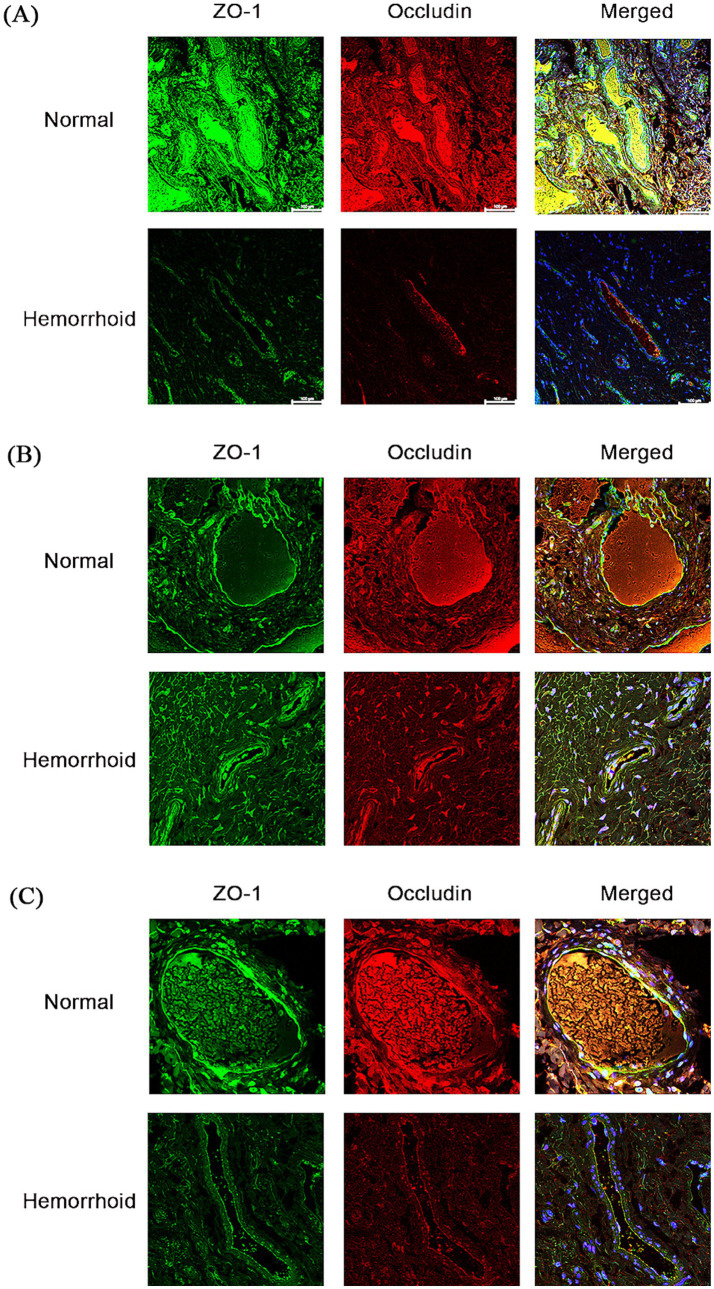
Immunofluorescence staining of ZO-1 and Occludin in human AVP. Compared with that in normal tissue, the fluorescence continuity of Occludin and ZO-1 in hemorrhoid tissue was interrupted, and the fluorescence intensity was weakened (**A–C** are three different groups of patients).

The immunofluorescence results of the human tissue specimens are shown in Figure 11. The results showed that the continuity of both ZO-1 and Occludin fluorescence in normal tissues was significantly greater than that in hemorrhoids tissues, and the fluorescence intensity was significantly greater than that in hemorrhoids tissues. It is suggested that the distribution of TJPs in the AVP region of hemorrhoids is more disordered than that in normal tissues.

## Discussion

Obesity induced by a high-fat diet is an independent risk factor for the development of hemorrhoids (9), and the development of hemorrhoids is closely related to VEBF injury. High concentrations of DCA induced by hyperlipidemia can reduce the expression of intestinal TJPs and alter the localization of TJPs (20), which in turn can disrupt the intestinal barrier (21). TJPs also exist in the endothelium of blood vessels. Therefore, an elevated DCA concentration may also have an injurious effect on the VEBF of AVP. Our study showed that SDC can alter the distribution of TJPs in AVP endothelial cells and impair barrier function in both cellular and animal models and that GYY4137 can ameliorate the damaging effect of SDC on VEBF.

In our study, a monolayer cell model of HUVECs was used to investigate the effect of SDC on VEBF, and we found that SDC could increase the permeability of the vascular endothelial monolayer. The lowest concentration (0.4 mmol/L) and the shortest duration of action (2 h) required to impair the monolayer barrier model were selected for SDC in our study, which is similar to the findings of previous reports of SDC at a concentration of 100 μM (22) and a 1 h duration of action (23). Similarly, Chen ZY et al. (19) showed that the application of 2.0 mmol/L SDC for 30 min could significantly affect the permeability of the Caco-2 monolayer cell model. So a certain concentration of SDC significantly increased the permeability of monolayer cells *in vitro*, both in epithelial and endothelial cell models. According to the Western blot results, the expression of Occludin and ZO-1 was slightly decreased in the SDC group, but the difference was not statistically significant. However, we considered that SDC possibly decreased the expression of TJPs; only the decrease in TJPs was not obvious due to the short duration of action of SDC. A prolonged duration of action may lead to a significant decrease in TJPs, as shown in Zeng HW et al.’s study (24). After treating Caco-2 cells with 0.25 mM and 0.3 mM SDC for 15 h, the Occludin levels decreased by 78 and 87%, respectively, relative to those in the control group.

Using an animal model, we assessed the effect of SDC on AVP permeability in mice via IgG immunohistochemistry. Macromolecular IgG, which is supposedly located in blood vessels, leaked significantly outside the vasculature in the SDC group, confirming that SDC resulted in increased AVP permeability and impaired VEBF in mice. Immunofluorescence staining of cellular and animal TJPs revealed that SDC can significantly alter the distribution of TJPs, demonstrating that SDC can indeed cause damage to AVP VEBF. Previous studies have shown that SDC can alter various structural elements of TPs, leading to injury of barrier function (25, 26). Wang ZW (22) et al. reported that DCA induces increased permeability in a Caco-2 monolayer cell model through activation of ERK1/2 and dynamic disruption of downstream TJ structures. Chen ZY (19) et al. demonstrated that SDC affects the expression and distribution of TJPs through the MLCK-P-MLC2 pathway, which in turn causes damage to the barrier function of intestinal epithelial cells. Our findings corroborate these results, confirming that SDC can have a similar damaging effect on the endothelium of AVP. We also applied immunohistochemical staining for MPO to evaluate the inflammation of AVP in mice. Neutrophils are important inducers of endothelial cell hyperpermeability (27), and MPO is a marker of leukocyte infiltration (28). We found that SDC could significantly exacerbate the aggregation of MPO and leukocytes in the vessel wall, which laterally indicated that DCA could somewhat aggravate the vascular inflammation of hemorrhoidal AVP.

Studies have shown that H2S can be involved in a variety of physiopathological processes, such as anti-inflammation (15), vasodilatation (16), and the promotion of healing of damaged tissues (29). Moreover, GYY4137 can inhibit the atherosclerotic modification of LDL (30), block monocyte adhesion caused by endothelial cell activation (31), and inhibit thrombosis and platelet aggregation (32). As a new H2S donor, GYY4137 can release H2S stably for a long time (17, 33). Chen SW et al. reported that GYY4137 could alleviate intestinal function injury by inhibiting the decrease in the expression and abnormal distribution of TJPs caused by endotoxin or TNF-*α* (17). Chen ZY et al. (19) found that GYY4137 can reduce intestinal barrier dysfunction caused by SDC by inhibiting the activation of the MLCK-P-MLC2 pathway. In our study, we found that GYY4137 could significantly reduce the increase in permeability of a HUVECs monolayer cell model induced by SDC and could significantly ameliorate the SDC-induced distribution disorder of TJPs at both the cellular and animal tissue levels. The increase in immunofluorescence intensity also suggested that GYY4137 could probably increase the expression of TJPs. These findings suggested that GYY4137 can have a similar protective effect on VEBF of AVP in addition to protecting intestinal epithelial function. We also found that GYY4137 can reduce vascular inflammation caused by SDC through immunohistochemical staining of MPO, which reflects the anti-inflammatory effect of GYY4137 on the AVP endothelium; its anti-inflammatory mechanism may be related to the direct inhibitory effect of H2S on the activation of NK-Κb (34), which warrants further exploration.

We subsequently explored the potential pathways and mechanisms underlying the effects of SDC and GYY4137 on the VEBF of AVP. Studies have shown that myosin light chain kinase (MLCK) plays an important role in regulating the distribution of TJPs. MLCK can phosphorylate myosin light chain 2 (MLC2) at threonine 18 and/or serine 19 (35), resulting in actin contraction and cytoskeletal remodeling, thereby pulling TJPs and causing their distribution to be disrupted (36–38). The results of our cellular studies showed that the expression of MLCK and P-MLC2 was significantly increased in the SDC group but was decreased when GYY4137 was added, suggesting that SDC can promote MLC2 phosphorylation by increasing the expression of MLCK, thus affecting the distribution of TJPs, while GYY4137 restored the distribution of TJPs by downregulating the phosphorylation of MLC2. However, it has been shown that, in addition to MLCK, the RhoA/ROCK pathway is involved in the phosphorylation of MLC2. RhoA can activate its downstream Rho-kinase (ROCK), which subsequently phosphorylates and inhibits myosin light chain phosphatase, leading to increased phosphorylation of MLC2 and contraction of actomyosin (39). This topic remains to be explored next.

We also performed immunofluorescence staining of TJPs in human tissue specimens. We found that, compared with that of normal AVP, the distribution of TJPs fluorescence in the AVP of hemorrhoidal patients was discontinuous, and the fluorescence intensity was significantly reduced. Pathological changes such as abnormal vascular wall dilatation, edema, and tension disorders in AVP of hemorrhoids[40] may be closely related to the distribution disorder and abnormal expression of TJPs, and these results are consistent with our cell and animal experiments.

VEBF disorders are associated with the pathogenesis of a variety of diseases, including cardiovascular disease (40), brain dysfunction (41), and lung disease (42). Mao et al. (43) reported that exosomal miR-375-3p increased the permeability of pulmonary vascular endothelial cells, contributing to the transendothelial migration of small-cell lung cancer cells. Liu MM et al. (44) found that the diammonium glycyrrhizinate lipid ligand ameliorated lipopolysaccharide-induced acute lung injury by modulating vascular endothelial barrier function. Zhang R et al. (45) found that activation of protease-activated receptor-2 inhibited VE-cadherin expression and impaired cardiovascular VEBF. Hemorrhoids, as vascular diseases, involve pathological changes such as dilatation of the AVP wall, abnormal tension, and thin-wall-like changes that are closely related to VEBF injury. A study by Qiao et al. (46) revealed that in a rat hemorrhoidal model, Sanhuang ointment could effectively reduce the vascular permeability of AVP and reduce the leakage of body fluids from vessels to tissues through its anti-inflammatory effect. Combined with our findings, we hypothesize that hyperlipidemia can increase the level of DCA in the blood, which leads to the disturbance of the distribution of TJPs among the endothelial cells of the AVP through the MLCK-P-MLC2 pathway, which may be accompanied by a decrease in the expression of TJPs, leading to VEBF injury of the hemorrhoidal AVP and thus contributing to the development of hemorrhoids.

However, there are several limitations. First, we did not investigate whether prolonged action of SDC on HUVECs could have a significant effect on ZO-1 and Occludin expression. Second, we did not investigare the long-term effects of GYY4137. Third, we failed to isolate the vascular components of animal tissues for Western blot analysis to detect the expression of TJPs. Fourth, whether H2S can produce protective effects in human tissues has not been investigated. All of the above factors need to be further explored.

In summary, we think that GYY4137 (H2S) can ameliorate SDC-induced VEBF injury of AVP in cellular models and animal tissues. It improves the distribution of TJPs by inhibiting the activation of the MLCK-P-MLC2 signaling pathway induced by SDC, which may be one of the potential mechanisms of its protective effect. Our study may lead to the development of treatments for hemorrhoids associated with hyperlipidemia.

## Data Availability

The raw data supporting the conclusions of this article will be made available by the authors, without undue reservation.
